# Diffuse Symptomatic Familial Gastric Polyposis Requiring Total Gastrectomy: A Complex Case Report

**DOI:** 10.3390/reports9020162

**Published:** 2026-05-21

**Authors:** Ivan Pesic, Ilija Golubovic, Milorad Pavlovic, Milica Nestorovic, Ivan Ilic

**Affiliations:** 1 Clinic for Digestive Surgery, University Clinical Center Nis, 18000 Nis, Serbia; golubovicilija@yahoo.com (I.G.); milica20@yahoo.com (M.N.); 2 Faculty of Medicine, University of Nis, 18000 Nis, Serbia; misapavlovicnis@gmail.com (M.P.); ilicko81@gmail.com (I.I.); 3 Clinic for Thoracic Surgery, University Clinical Center Nis, 18000 Nis, Serbia; 4 Center for Pathology, University Clinical Center Nis, 18000 Nis, Serbia

**Keywords:** familial gastric polyposis, gastrectomy, intestinal metaplasia, hereditary cancer, case report

## Abstract

**Background and Clinical Significance**: Familial gastric polyposis is a rare condition associated with an increased risk of malignant transformation, particularly in patients with a strong family history of gastrointestinal malignancies. **Case Presentation**: We report the case of a 46-year-old female presenting with severe epigastric pain, persistent vomiting, and significant weight loss. Endoscopic and histopathological evaluation confirmed diffuse fundic gland polyposis with intestinal metaplasia involving the entire gastric mucosa. Given the extensive disease, pronounced symptoms, and significant familial cancer burden, the patient underwent total gastrectomy with Roux-en-Y esophagojejunostomy. The postoperative course was uneventful, with satisfactory recovery and favorable functional outcomes during follow-up. This case highlights the clinical challenges associated with diffuse symptomatic familial gastric polyposis and underscores the importance of timely surgical intervention in high-risk patients. **Conclusions**: Due to clinical complexity of such presentations, detailed case descriptions remain important for guiding clinical practice.

## 1. Introduction and Clinical Significance

Familial gastric polyposis represents a rare clinical entity characterized by the presence of multiple gastric polyps, often occurring in the context of hereditary syndromes and associated with an increased risk of gastric carcinoma [1,2]. Although fundic gland polyps are generally considered benign lesions [3,4], their clinical significance increases when they occur in a familial setting, particularly in the presence of associated histo-pathological changes such as intestinal metaplasia [5,6].

These patients require careful clinical evaluation, as the combination of genetic predisposition and mucosal alterations may be associated with a higher long-term risk of malignant transformation [7,8]. Early recognition and appropriate risk stratification are therefore essential, especially in symptomatic individuals or those with a strong family history of gastrointestinal malignancies [9,10].

In most cases, management is based on endoscopic surveillance and histological monitoring. However, in advanced or diffuse forms of disease, when the entire gastric mucosa is involved and symptoms are present, endoscopic approaches may be insufficient. In such cases, surgical treatment, including total gastrectomy, remains the definitive therapeutic option [11].

Familial gastric polyposis is also frequently associated with hereditary cancer syndromes [12,13] in which cumulative lifetime risk of gastric carcinoma may be significantly increased. While standardized guidelines for timing of surgical intervention are limited due to the rarity of the condition, management decisions must be individualized, taking into account symptom severity, extent of disease, and familial cancer burden. Therefore, well-documented case reports remain important due to the clinical complexity and variability of such presentations.

The aim of this case report is to describe the clinical presentation, diagnostic evaluation, surgical management, and postoperative outcome of a patient with diffuse symptomatic familial gastric polyposis requiring total gastrectomy, and to highlight the importance of individualized treatment strategies in rare and complex gastric polyposis cases.

## 2. Case Presentation

A 46-year-old female was admitted to the Clinic for Digestive Surgery, University Clinical Center Nis, for surgical treatment of previously diagnosed familial gastric polyposis. The diagnosis had been established by esophagogastroduodenoscopy and confirmed histologically. The patient was evaluated for a possible hereditary gastrointestinal polyposis syndrome. However, no genetic testing was performed, and there was no clinical or endoscopic evidence suggestive of familial adenomatous polyposis (FAP) or gastric adenocarcinoma and proximal polyposis of the stomach (GAPPS).

The patient presented with severe epigastric pain, inability to tolerate food and fluids, and frequent vomiting of undigested food. She reported a weight loss of approximately 7–10 kg over the preceding three months.

One month prior to admission, esophagogastroduodenoscopy revealed diffusely distributed and extensive polypoid lesions involving the entire gastric mucosa, with more than 50 lesions covering the fundus, corpus, and partially the antrum. The polyps varied in size from a few millimeters up to approximately 1 cm and were diffusely distributed throughout the gastric mucosa, predominantly involving the fundus and corpus, with relative sparing of the antral region. Multiple biopsies were obtained, and histopathological findings indicated fundic gland polyposis with incomplete-type intestinal metaplasia.

The patient’s medical history included a 20-year history of smoking (approximately five cigarettes daily), with no alcohol consumption. Family history was significant for malignancies: her mother died of pancreatic and colorectal cancer at the age of 65, while her older sister died of gastric cancer at the age of 51. Both had documented polyposis syndromes. Genetic testing was not performed in this case due to the absence of specific clinical indications for a defined hereditary polyposis syndrome, and no molecular confirmation was available.

On admission, the patient weighed 55 kg with a body mass index (BMI) of approximately 18.0 kg/m^2^, indicating a borderline underweight nutritional status. Laboratory findings were as follows: leukocytes 6.3 × 10^9^/L, erythrocytes 3.86 × 10^12^/L, hemoglobin 118 g/L, hematocrit 0.349, platelets 171 × 10^9^/L, and glucose 3.8 mmol/L, with other biochemical parameters within normal limits. The patient’s nutritional status was moderately compromised due to prolonged inadequate oral intake, which further supported the decision for definitive surgical management. Helicobacter pylori was not detected in the available biopsy and resection specimen. The patient had no history of long-term proton pump inhibitor (PPI) use.

The indication for total gastrectomy was based on diffuse involvement of the entire gastric mucosa, severe and progressive symptoms including inability to tolerate oral intake and significant weight loss, and the presence of multifocal intestinal metaplasia in the setting of a strong familial history of gastric and colorectal malignancies. Given the extensive and non-resectable nature of the disease, endoscopic treatment or surveillance was not considered appropriate, and radical surgical treatment was indicated.

Following adequate preoperative preparation, the patient underwent open surgical treatment, including total gastrectomy, cholecystectomy, total omentectomy, and radical lymphadenectomy. Reconstruction was performed using Roux-en-Y esophagojejunostomy (termino-lateral) with a circular stapler (EEA 25 mm). Duodenal closure was achieved using a linear stapler (GIA 80 mm). A nasojejunal feeding tube and abdominal drainage were placed.

**Figure 1:**
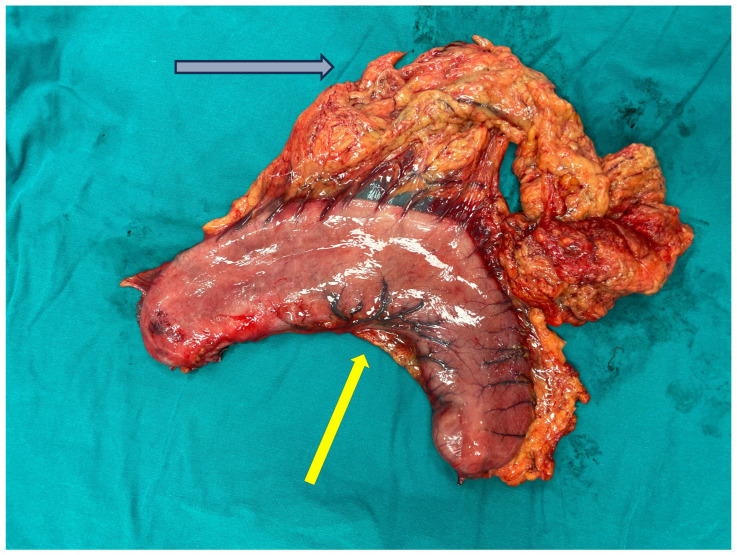
Macroscopic appearance of the resected stomach (yellow arrow) following total gastrectomy with attached omentum (grey arrow). The specimen demonstrates the external gastric wall without focal tumor mass, consistent with diffuse pathological involvement.

**Figure 2:**
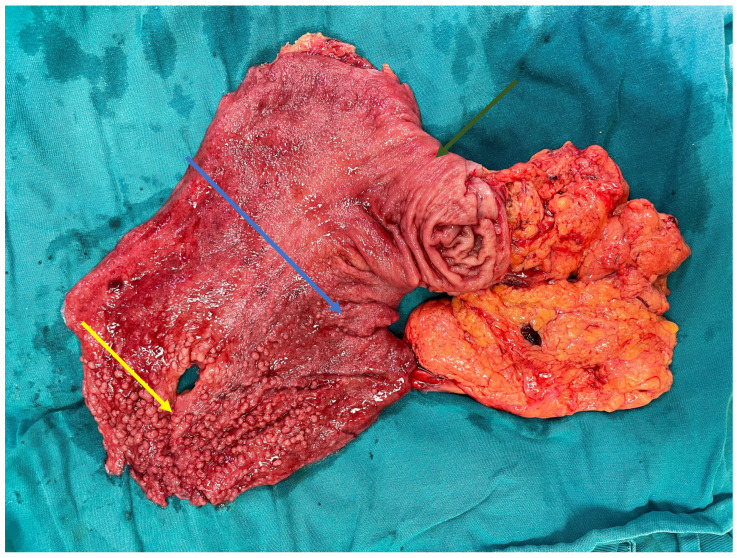
Opened gastric specimen demonstrating diffuse involvement of the gastric mucosa by multiple fundic gland polyps (yellow arrow), consistent with familial gastric polyposis. The lesions are distributed throughout the fundus (yellow arrow) and corpus (blue arrow) with relative preservation of the antral region (green arrow). Arrows indicate representative polypoid lesions.

**Figure 3:**
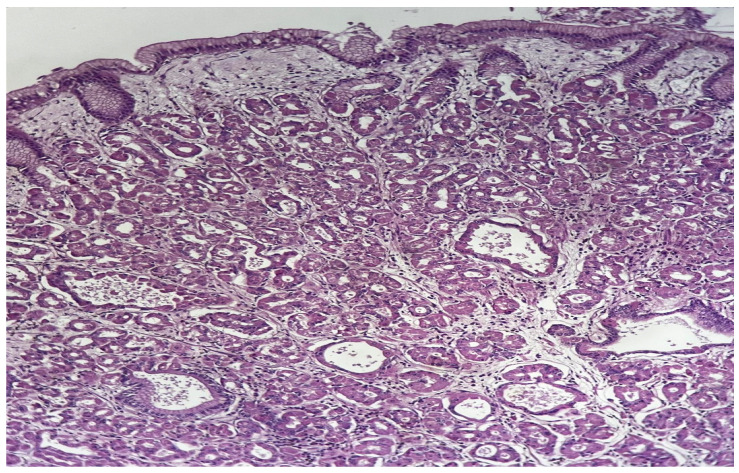
Histopathological findings of fundic gland polyp showing cystically dilated glands lined by chief cells, parietal cells, and mucinous foveolar epithelium. Hyperplastic parietal cells with characteristic apocrine snouting are present (H&E stain, ×100).

The macroscopic appearance of the resected specimen is shown in Figure 1 and Figure 2. Representative histopathological findings are presented in Figure 3.

The postoperative course was uneventful. No blood transfusions were required. Intestinal peristalsis was established on postoperative day 4, and enteral feeding via the nasojejunal tube was initiated without complications.

On postoperative day 9, contrast radiography confirmed a patent esophagojejunal anastomosis without leakage. The nasojejunal tube was removed, and oral intake was initiated successfully. Drains and sutures were removed prior to discharge.

Laboratory findings at discharge showed mild postoperative anemia (hemoglobin 99 g/L) and hypoalbuminemia (32 g/L), while other parameters remained within acceptable postoperative ranges.

Final histopathological examination confirmed diffuse fundic gland polyposis involving the entire gastric mucosa, associated with chronic atrophic gastritis (grade II, active) and multifocal intestinal metaplasia. No evidence of dysplasia or invasive carcinoma was identified. Intestinal metaplasia represented a preneoplastic mucosal alteration.

Histopathological examination of the gastroesophageal junction and distal esophagus revealed chronic inactive gastritis without evidence of intestinal metaplasia, dysplasia, or neoplastic changes. The esophagojejunal junction showed chronic inactive atrophic gastritis.

No significant histological differences were observed between polyps located in the gastric fundus and corpus, as both regions demonstrated similar features of fundic gland polyposis. The antral mucosa was relatively spared, without polypoid changes, but showed chronic atrophic gastritis with multifocal intestinal metaplasia. The intervening non-polypoid gastric mucosa showed chronic atrophic gastritis with multifocal areas of both complete and incomplete intestinal metaplasia.

The proximal duodenum was histologically unremarkable, with preserved mucosal architecture and no evidence of polypoid, dysplastic, or inflammatory changes. Helicobacter pylori status was not documented in the available histopathological examination.

At follow-up visits (2 weeks, 1 month, and 3 months postoperatively), the patient reported no difficulties with food intake or digestion, with occasional episodes of diarrhea. She gained 5 kg in body weight.

At 6-month follow-up, the patient remained asymptomatic. One year after surgery, endoscopic evaluation showed normal findings. Fourteen months postoperatively, she underwent surgery for a perianal fistula, with prior colonoscopy showing no abnormalities. At 18 months follow-up, the patient remained symptom-free.

## 3. Discussion

Familial gastric polyposis is a clinically relevant condition, particularly in patients with a strong family history of gastrointestinal malignancies [1,2,12,13]. Although fundic gland polyps are generally considered benign lesions, their occurrence in a familial setting, especially when associated with intestinal metaplasia, may be linked to an increased risk of malignant transformation [3,6]. This emphasizes the importance of careful risk stratification and long-term surveillance in affected patients.

In the present case, several high-risk features were present simultaneously, including diffuse involvement of the entire gastric mucosa, significant symptom burden with severe functional impairment, and a strong family history of gastric and colorectal malignancies [7,8,14]. The combination of these factors places the patient in a high-risk clinical category, where conservative or endoscopic management is often insufficient. In such scenarios, early recognition and timely decision-making are crucial for optimal outcomes.

The clinical significance of this case lies in the combination of diffuse gastric involvement, severe symptom burden with marked functional impairment, and a strong familial history of gastrointestinal malignancies, which collectively necessitated surgical management. This constellation of findings highlights the importance of individualized decision-making in complex gastric polyposis cases. This case further emphasizes the need for a patient-centered approach in similar complex clinical scenarios.

Endoscopic surveillance plays an essential role in the early detection and monitoring of gastric polyposis, particularly in patients with hereditary predisposition [9,10,12,13]. However, when disease is extensive and involves the entire gastric mucosa, as in this case, endoscopic treatment options are limited. Total gastrectomy with Roux-en-Y reconstruction remains the definitive therapeutic approach in such advanced or diffuse presentations [11,15]. Reported outcomes following this procedure are generally favorable, with acceptable morbidity and good long-term functional adaptation, which is consistent with the postoperative course observed in our patient [16]. Current guidelines support individualized management in extensive gastric polyposis, particularly when diffuse mucosal involvement and high-risk clinical features are present, as endoscopic approaches are limited in such cases.

The present case is notable due to the coexistence of diffuse gastric involvement, pronounced clinical symptoms, and strong familial clustering of gastrointestinal malignancies. While each of these factors alone may not necessarily indicate radical surgical treatment, their combination significantly increases clinical concern and supports an individualized therapeutic strategy. Such presentations are uncommon in routine clinical practice and often require deviation from standard stepwise management algorithms in favor of definitive surgical intervention.

Similar cases of extensive gastric polyposis requiring surgical treatment have been reported, particularly in patients with suspected or confirmed hereditary cancer syndromes [11,15,16]. These patients frequently present with progressive gastrointestinal symptoms, nutritional impairment, and variable degrees of mucosal involvement. However, the extent of disease distribution and timing of surgical intervention differ across reported cases. Histopathological evaluation remains essential not only for confirming the diagnosis but also for excluding dysplasia or early malignant transformation, especially in the presence of intestinal metaplasia.

Compared to previously published cases, our patient demonstrated a more pronounced symptom profile and complete gastric involvement, which strongly influenced the decision for early radical surgical management. This highlights the importance of individualized treatment strategies rather than rigid adherence to standardized protocols in selected high-risk patients.

Although fundic gland polyposis is generally considered a benign entity, accumulating evidence suggests that in the context of hereditary predisposition and extensive mucosal involvement, there may be an increased risk of dysplastic or neoplastic progression. In this case, the combination of diffuse disease, significant symptom burden, and strong familial cancer history collectively justified an aggressive surgical approach. These findings are in line with current literature emphasizing that management decisions should be tailored to individual risk profiles, with total gastrectomy representing a valid and effective option in carefully selected patients.

This case emphasizes that decision-making in familial gastric polyposis should be individualized, integrating clinical presentation, histopathological findings, and familial risk factors, rather than relying solely on endoscopic findings.

## 4. Conclusions

Familial gastric polyposis with associated risk factors requires timely recognition and appropriate management. Total gastrectomy represents a safe and effective treatment option in patients with diffuse disease and high malignant potential. Patients with pronounced symptoms and significant familial cancer burden represent a particularly important subgroup requiring early and aggressive management.

## Data Availability

The original contributions presented in this study are included in the article. Further inquiries can be directed to the corresponding author.
